# 2, 3, 7, 8‐Tetrachlorodibenzo‐*p*‐dioxin promotes endothelial cell apoptosis through activation of EP3/p38MAPK/Bcl‐2 pathway

**DOI:** 10.1111/jcmm.13265

**Published:** 2017-07-12

**Authors:** Yu Yu, Qian Liu, Shumin Guo, Qianqian Zhang, Juan Tang, Guizhu Liu, Deping Kong, Juanjuan Li, Shuai Yan, Ruiguo Wang, Peilong Wang, Xiaoou Su, Ying Yu

**Affiliations:** ^1^ Department of Pharmacology Tianjin Medical University Tianjin China; ^2^ Department of Pediatric Cardiology Xinhua Hospital affiliated to Shanghai Jiao Tong University School of Medicine Shanghai China; ^3^ Key Laboratory of Food Safety Research Institute for Nutritional Sciences Shanghai Institutes for Biological Sciences Chinese Academy of Sciences Shanghai China; ^4^ Institute of Quality Standards and Testing Technology for Agro‐products Chinese Academy of Agricultural Sciences Beijing China

**Keywords:** dioxin, endothelial cell, apoptosis, EP3, p38 MAPK, Bcl‐2

## Abstract

Endothelial injury or dysfunction is an early event in the pathogenesis of atherosclerosis. Epidemiological and animal studies have shown that 2, 3, 7, 8‐tetrachlorodibenzo‐*p*‐dioxin (TCDD) exposure increases morbidity and mortality from chronic cardiovascular diseases, including atherosclerosis. However, whether or how TCDD exposure causes endothelial injury or dysfunction remains largely unknown. Cultured human umbilical vein endothelial cells (HUVECs) were exposed to different doses of TCDD, and cell apoptosis was examined. We found that TCDD treatment increased caspase 3 activity and apoptosis in HUVECs in a dose‐dependent manner,at doses from 10 to 40 nM. TCDD increased cyclooxygenase enzymes (COX)‐2 expression and its downstream prostaglandin (PG) production (mainly PGE_2_ and 6‐keto‐PGF_1α_) in HUVECs. Interestingly, inhibition of COX‐2, but not COX‐1, markedly attenuated TCDD‐triggered apoptosis in HUVECs. Pharmacological inhibition or gene silencing of the PGE_2_ receptor subtype 3 (EP3) suppressed the augmented apoptosis in TCDD‐treated HUVECs. Activation of the EP3 receptor enhanced p38 MAPK phosphorylation and decreased Bcl‐2 expression following TCDD treatment. Both p38 MAPK suppression and Bcl‐2 overexpression attenuated the apoptosis in TCDD‐treated HUVECs. TCDD increased EP3‐dependent Rho activity and subsequently promoted p38MAPK/Bcl‐2 pathway‐mediated apoptosis in HUVECs. In addition, TCDD promoted apoptosis in vascular endothelium and delayed re‐endothelialization after femoral artery injury in wild‐type (WT) mice, but not in EP3^−/−^ mice. In summary, TCDD promotes endothelial apoptosis through the COX‐2/PGE_2_/EP3/p38MAPK/Bcl‐2 pathway. Given the cardiovascular hazard of a COX‐2 inhibitor, our findings indicate that the EP3 receptor and its downstream pathways may be potential targets for prevention of TCDD‐associated cardiovascular diseases.

## Introduction

Endothelium plays an essential role in regulation of vascular homeostasis and thrombosis, vascular tone and redox balance. Endothelial cell (EC) dysfunction or apoptosis facilitates atherosclerosis 1, 2, 3, 4, 5. Long‐term exposure to environmental toxicants and pharmaceutical chemicals can result in endothelial injury 6, 7, 8, 9.

2,3,7,8‐Tetrachlorodibenzo‐*p*‐dioxin (TCDD) is the most toxic congener of the dioxins, whose toxicological effects are mediated by interaction with the aryl hydrocarbon receptor (AhR), an orphan nuclear receptor 10. Emerging epidemiological studies have shown that TCDD exposure increases the risk of cardiovascular diseases. For example, TCDD exposure leads to a higher incidence of hyperlipidaemia, as well as increased atherosclerotic plaques and intima‐media thickness 11, 12. In animal experiments, TCDD treatment has been shown to enhance atherosclerotic lesions in ApoE^−/−^ mice 13, 14. However, the underlying mechanisms of TCDD‐associated cardiovascular diseases are not fully understood. Previous studies have reported that TCDD causes apoptosis in many types of cells, including chondrocytes 15, lymphoblastic T cells 16 and pheochromocytoma cells 17. Whether TCDD exposure promotes endothelial cell apoptosis has not been determined.

Prostaglandins (PGs) are implicated in the regulation of endothelial homeostasis 18. Cyclooxygenase enzymes (COX‐1 and COX‐2) are the rate‐limiting enzymes for the conversion of arachidonic acid (AA) to downstream PGs. COX‐2 inhibitors have been shown to improve vascular function in diabetic rats by suppressing vascular endothelial growth factors 19, meanwhile high glucose promotes human umbilical vein endothelial cells (HUVECs) apoptosis by up‐regulating of COX‐2 through activation of NF‐кB signalling 20. Interestingly, TCDD exposure increases COX‐2 mRNA levels in HUVECs 6, mouse lung endothelial cells (MLECs) 6, rat hepatocytes 21 and mouse fibroblasts 22. We envision that TCDD exposure may elicit EC apoptosis by inducing COX‐2 expression and its downstream PG products. Accumulated evidence has shown that COX‐2‐derived PGs and downstream PG receptors play important roles in cellular apoptosis. For example, increased mast cell apoptosis by PGE_2_ is dependent on an increase in E‐prostanoid receptor (EP) subtype 3 (EP3) activation, intracellular calcium release and calmodulin‐dependent kinase II and MAPK activation 23. The role of PG and its receptors in TCDD‐caused endothelial cell apoptosis has yet to be determined.

This study aimed to determine whether TCDD causes apoptosis in endothelial cells and to explore the potential mechanisms involved. Our data showed that HUVEC apoptosis caused by TCDD depends on COX‐2 activity. Inhibition of COX‐2 and the PGE_2_ receptor subtype EP3 suppressed apoptosis in TCDD‐treated HUVECs. Moreover, activation of the EP3 receptor increased Rho activity and p38 MAPK phosphorylation and decreased Bcl‐2 expression in TCDD‐treated HUVECs, while suppression of Rho and p38 MAPK activity abrogated increased apoptosis in HUVECs stimulated by TCDD. Additionally, TCDD exposure reduced EP3 receptor‐dependent re‐endothelialization of femoral arteries after targeted injury in mice.

## Methods

### Animals

EP3 knockout (EP3^−/−^) mice and wild‐type (WT) C57BL/6 were used 24. Femoral arteries of 10‐ to 12‐week‐old male mice were injured using a wire 0.38 mm in diameter (Cook Group Inc., Bloomington, IN, USA). Then, the mice were injected with 10 μg TCDD/kg bodyweight intraperitoneally every other day. On the tenth day, the injured femoral arteries were collected for further examination. All animal procedures were approved by the institutional Animal Care and Use Committee of the Institute for Nutritional Sciences, Chinese Academy of Sciences.

### Cell culture and drug preparation

HUVECs were obtained from the Cell Resource Center of Shanghai Institutes for Biological Sciences, Chinese Academy of Sciences. Cells were cultured in RPMI 1640 medium (Invitrogen, Carlsbad, CA, USA) supplemented with 10% foetal bovine serum (FBS, HyClone), streptomycin (100 mg/ml; Sigma‐Aldrich, Saint Louis, Missouri, USA) and penicillin (100 units/ml; Sigma‐Aldrich) and maintained at 37 °C under a humidified atmosphere containing 5% CO_2_. The cells were seeded into 6‐ or 96‐well plates, stimulated by various drugs for different desired time intervals and then collected for further analysis. The drugs used to stimulate the cells were as follows: SC‐560 (COX‐1 inhibitor); NS‐398 (COX‐2 inhibitor); SC51322 (EP1 antagonist); AH6809 (EP2 antagonist); L798106 (EP3 antagonist); L‐161982 (EP4 antagonist); PD98059 (ERK inhibitor); SB203580 and SB202190 (p38 MAPK inhibitors); SP600125 (JNK inhibitor); Y27632 (ROCK inhibitor); cicaprost, PGE_2_, and AA (Cayman Chemical, Ann Harbor, MI, USA); TCDD (Cambridge Isotope Laboratories, Tewksbury, MA, USA).

### Cell viability assay

HUVECs were seeded into 96‐well plates at the same cell number per well (5 × 10^3^/well). Once the cells reached 50% confluence, they were pre‐treated with various inhibitors for 24 hrs and incubated with TCDD at indicated concentrations for an additional 24 hrs. At the end of each culture, the medium was removed and replaced with 100 μl fresh medium and 10 μl Cell Counting Kit‐8 (CCK‐8) Assay Kit reagent (Dojindo, Kumamoto, Japan). Plates were incubated at 37°C for 2 hrs. Next, absorbance was measured at 450 nm using a SpectraMax 190 microplate reader (Molecular Devices, Sunnyvale, CA, USA). The reference wavelength was 600 nm. Background absorbance (from wells without cells) was subtracted from all values, and data are presented as relative values to controls as previously described 25. All experiments were performed in triplicate with 3–5 repeats per culture.

### Cell apoptosis detection

Apoptosis was assessed with an Annexin V‐FITC/Propidium Iodide (PI) apoptosis detection kit (Dojindo) using a BD FACSAria flow cytometer (BD Biosciences, San Jose, CA, USA). Briefly, HUVECs were seeded into 6‐well plates and incubated with various inhibitors for 24 hrs and then stimulated with TCDD for an additional 24 hrs. The cells were then harvested and washed once in phosphate‐buffered saline (PBS) and once in 1× binding buffer. After resuspension in 1× binding buffer, we added 5 μl Annexin V to 100 μl cell suspension. Next, the mixture was incubated for 15 min. at room temperature, washed and resuspended in 1× binding buffer. After addition of 5 μl PI dye, HUVECs were analysed by flow cytometry. In addition, caspase 3 activity was assayed using a Caspase 3 Activity Assay Kit (Beyotime, Haimen, China) according to the manufacturer's instructions. Data are shown as relative values to controls and representative of at least three independent experiments 20.

### DAPI staining

The procedure was performed as described previously 26. In brief, HUVECs were washed twice with PBS. Next, the cells were fixed with 3.7% paraformaldehyde for 10 min. at room temperature and washed twice with PBS. Afterwards, the cells were stained with DAPI (0.5 g/ml) for 1 hr in the dark. The stained cells were washed with PBS and examined by fluorescence microscopy. Apoptotic cells were identified by condensation and fragments of nuclei.

### RNA interference and Bcl‐2 overexpression

Cells were transiently transfected with 100 pmol AhR nuclear translocator (ARNT), EP1‐4 receptor or scrambled siRNA per well in 6‐well plates, or 5 pmol EP1‐4 receptor or scrambled siRNA per well in 96‐well plates using Lipofectamine 2000 reagent (Invitrogen) according to the manufacturer`s instruction. Three pairs of siRNA specific sequences for ARNT and EP1‐4 mRNA were designed by Shanghai GenePharma Co., Ltd. (Shanghai, China). The most effective sequences are shown in Table S1. In addition, cells were seeded into 6‐well plates or 96‐well plates, and transfected with Bcl‐2‐pcDNA 3.1 plasmid (Addgene) or vector using Lipofectamine 2000 reagent (Invitrogen). All analyses were performed for 24 hrs after transfection and TCDD stimulation.

### RNA extraction and quantitative real‐time polymerase chain reaction (qRT‐PCR)

Total RNAs were isolated from HUVECs using TRIzol reagent (Invitrogen) according to the manufacturer's instructions and treated with DNase I (Promega, Madison,WI, USA). RNA concentration and purity were assessed using a NanoDrop 2000 spectrophotometer (Thermo Fisher Scientific, Wilmington, USA). RNA (2.0 μg) was reverse‐transcribed with Reverse Transcription Reagent kits and oligo (dT) 18 primers (Takara Bio, Inc. Dalian, China) as recommended. qRT‐PCR was performed using a CFX96™ real‐time PCR detection system (Bio‐Rad, Hercules, California, USA) and iQ™ SYBR Green Supermix (Bio‐Rad) as described by the manufacturer. Gene expression was normalized to the internal control, *GAPDH* and presented as relative levels calculated by the 2^ΔΔ^Ct method 27. All specific primers for the genes investigated are listed in Table S1.

### Western blotting

Proteins from total cell lysates were separated by 10% SDS‐PAGE and transferred electrophoretically onto Immobilon‐P polyvinylidene difluoride (PVDF) membranes (Merck Millipore, Darmstadt, Germany). The membranes were probed with rabbit anti‐COX‐1 polyclonal antibody (1:1,000; Cayman Chemical), rabbit anti‐COX‐2 polyclonal antibody (1:200; Cayman Chemical), rabbit anti‐p38 MAPK monoclonal antibody (1:1,000; Cell Signaling Technology, Danvers, MA, USA, catalogue no. 9212S), rabbit anti‐p‐p38 MAPK (T180/Y182) monoclonal antibody (1:1,000; Cell Signaling Technology, catalogue no. 9211S), mouse anti‐Bcl‐2 monoclonal antibody (1:500; Proteintech, Rosemont, IL, USA), mouse anti‐RhoA monoclonal antibody (1:500; Santa Cruz Biotechnology, Ann Harbor, MI, USA) or mouse anti‐tubulin monoclonal antibody (1:5,000, Cell Signaling Technology) followed by either anti‐rabbit or anti‐mouse IgG secondary antibodies conjugated to horseradish peroxidase (1:4000; Proteintech) and detection with the enhanced chemiluminescence (ECL) system (Pierce, Rockford, IL, USA). The films were scanned by the ImageQuant LAS 4000 mini biomolecular imager (GE Healthcare, Chicago, Illinois, USA). The relative protein density was quantified by ImageJ 1.44 (NIH, Bethesda, Maryland, USA).

### Rho GTPase activity assay

HUVECs were grown to confluence on 15 cm^2^ dishes, and equal numbers of serum‐starved cells were incubated with TCDD (40 nM) or DMSO in RPMI 1640 for 3 min. at 37°C. The cells were washed with cold PBS and then harvested in ice‐cold lysis buffer. RhoA activation assay kits were purchased from Cell Biolabs (San Diego, CA, USA) and used to detect active RhoA according to the manufacturer's protocol.

### PG extraction and analysis

HUVECs were treated with DMSO, 20 μM NS‐398, 40 nM TCDD or a combination of TCDD with 20 μM NS‐398 for 24 hrs, after which the medium was changed to the medium containing fresh FBS and 30 μM arachidonic acid (AA) for 15 min. The medium was collected for PG production analysis with liquid chromatography/mass spectrometry/mass spectrometry (LC/MS/MS; Agilent Technologies, Santa Clara, California, USA) and normalized to total protein. The detailed procedure was routinely performed in our laboratory 28.

### Femoral artery wire injury model

The femoral artery wire injury model has been described previously elsewhere 28. In brief, mice were anesthetized with isoflurane during surgery. The left femoral arteries were exposed by blunt dissection and monitored under a surgical microscope. After the distal artery was encircled with a 5‐0 silk suture, a vascular clamp was placed near the inguinal ligament, and a guide wire (0.38 mm in diameter; Cook Group Inc., Bloomington, IN, USA) was inserted into the arterial lumen through an arteriotomy made in the distal perforating branch. The guide wire was left in place for 3 min. to denude the artery. Next, the wire was removed, and the silk suture was released to restore blood flow. The skin incision was closed with a 6‐0 silk suture. Mice were allowed to recover, and femoral arteries were harvested 10 days post‐injury.

### TUNEL and Evans blue staining

To examine whether TCDD is involved in apoptosis and re‐endothelialization *in vivo*, we employed TUNEL staining and the Evans blue dye methods 29, 30. Briefly, the mice were injected intravenously with 0.5 ml of 0.5% Evans blue dye (Sigma‐Aldrich) in PBS on the tenth day after vascular injury. The injured femoral arteries were then incised longitudinally to expose the luminal surface and fixed in 4% polyformaldehyde solution for 5 min. Planimetric analysis was performed to calculate the ratio of the re‐endothelialized area, defined as an area not stained with Evans blue relative to the entire luminal surface area of injured arteries. In addition, frozen sections of the femoral arteries were prepared; then, DNA fragments were detected using the terminal deoxynucleotidyl transferase‐mediated dUTP nick end labelling (TUNEL) assay with an Apoptosis Detection Kit (Yesen, Shanghai, China) according to the manufacturer's protocols.

### Statistical analysis

Data are expressed as means ± S.E.M. *P* values were calculated by a two‐tailed Student's *t*‐test or one‐ or two‐way anova with Bonferroni's *post hoc* analyses as appropriate (GraphPad Prism 5.0 software). *P* < 0.05 was considered statistically significant.

## Results

### TCDD promotes apoptosis in HUVECs

**Figure 1:**
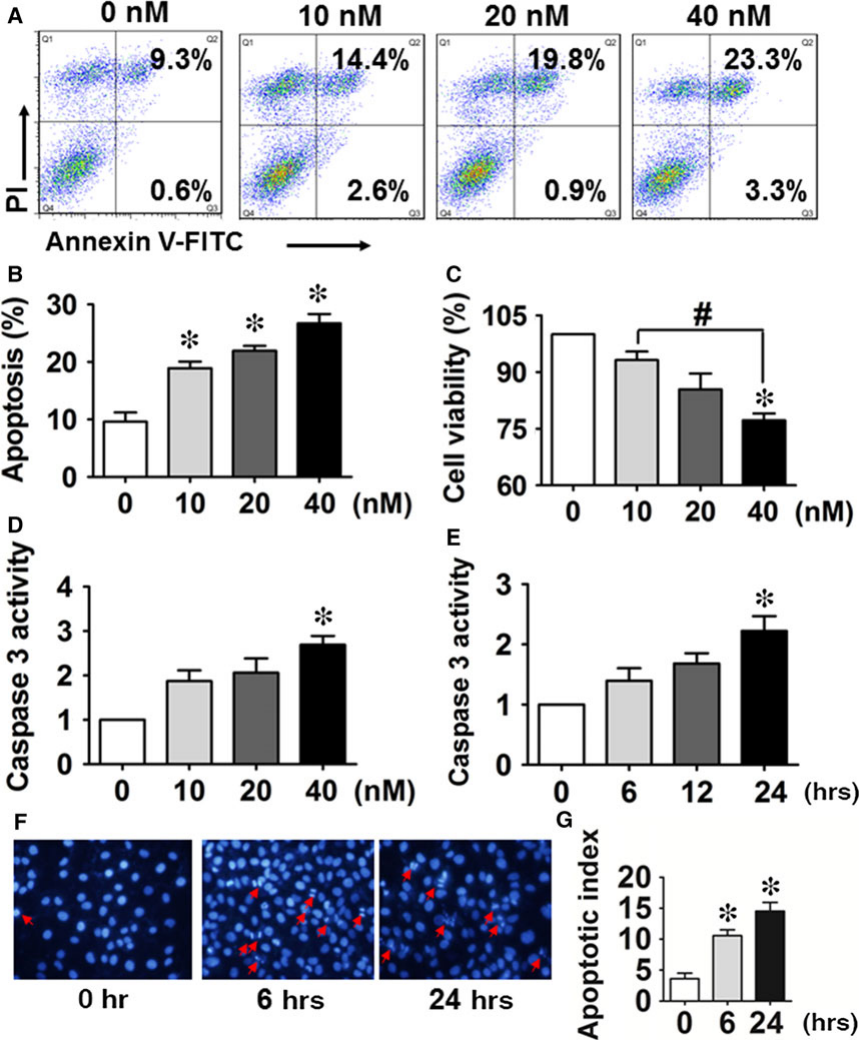
2, 3, 7, 8‐tetrachlorodibenzo‐*p*‐dioxin (TCDD) exposure increases apoptosis in human umbilical vein endothelial cells (HUVECs). (**A**) Annexin V‐FITC/PI staining of HUVECs treated with different concentrations of TCDD for 24 hrs was assessed by flow cytometry. The percentages of early and late apoptotic cells are presented in the lower right and upper right quadrants, respectively. (**B**) Columns represent the average proportions of apoptotic cells. *P < 0.05 *versus* the 0 nM group; *n* = 3. (**C**) Cell viability in HUVECs exposed to 0, 10, 20 and 40 nM TCDD was analysed by the CCK‐8 assay kit. *P < 0.05 *versus* the 0 nM group, ^#^ *P* < 0.05 *versus* the 10 nM group; *n* = 3. (**D–E**) The effects of various TCDD doses (0–40 nM) and duration of exposure (at 40 nM) on caspase 3 activity in HUVECs. *P < 0.05 *versus* the 0 nM group; *n* = 3. (**F**) Apoptotic morphology of HUVECs incubated with 40 nM TCDD for 6 or 24 hrs after DAPI staining. Apoptotic cells were identified by condensation and fragmentation of nuclei. (**G**) The apoptotic index (AI) was calculated as the percentage of apoptotic nuclei per total nuclei number per field. Values represent mean ± SEM of four independent experiments, *P < 0.05 *versus* the 0 nM group; *n* = 4.

We first examined the apoptosis, cell viability, caspase 3 activity and apoptotic morphology of HUVECs in response to TCDD stimulation. As shown in Figure 1A and B, PI—an indicator of late apoptosis—and Annexin V‐FITC co‐staining was performed to examine the apoptosis. We observed that TCDD caused apoptosis in a dose‐dependent manner (*P* < 0.05), and the apoptosis of the group treated with 40 nM TCDD (26.24 ± 2.19%) was nearly threefold (*P* < 0.05) higher than that of the control group (9.67 ± 1.55%). We also found that 40 nM TCDD significantly decreased (*P* < 0.05) the cell viability from 100% to 76.98% (Fig. 1C). As caspase 3 activation is an indicator of cell apoptosis, we examined the caspase 3 activity in HUVECs treated by TCDD at different concentrations and exposure times, and observed more than twofold increases of caspase 3 activity (*P* < 0.05; Fig. 1D and E) at 40 nM TCDD after 24 hrs. Meanwhile, TCDD promoted condensation and fragmentation of nuclei in HUVECs as seen by DAPI staining (*P* < 0.05; Fig. 1F and G), further confirming that TCDD leads to apoptosis in HUVECs.

### COX‐2 is involved in TCDD‐caused apoptosis of HUVECs

**Figure 2:**
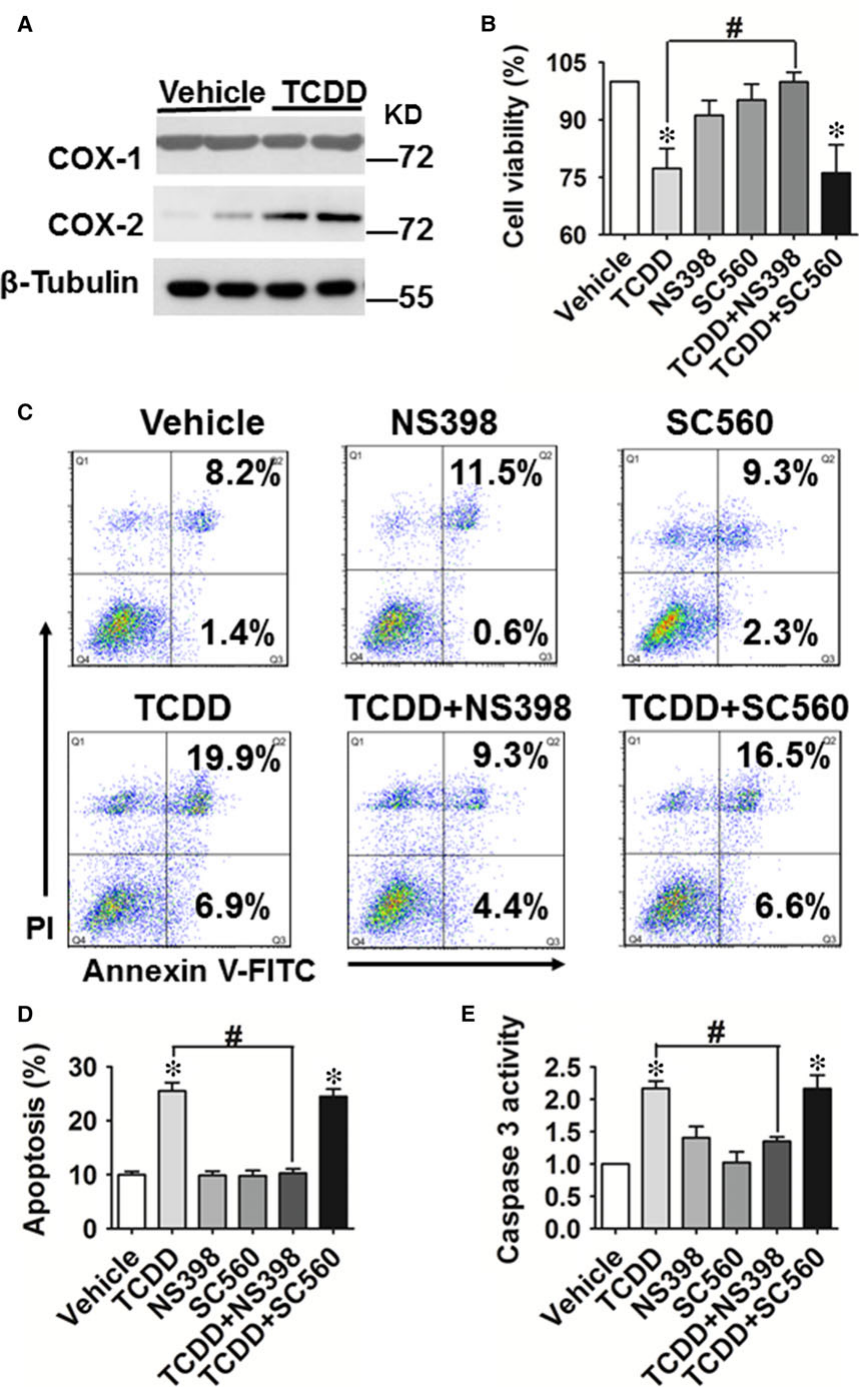
COX‐2 is involved in 2, 3, 7, 8‐tetrachlorodibenzo‐*p*‐dioxin (TCDD)‐caused apoptosis in human umbilical vein endothelial cells (HUVECs). (**A**) Protein levels of COX‐1 and COX‐2 in HUVECs treated with or without TCDD (40 nM) for 24 hrs were analysed by Western blot. (**B**) Cell viability in HUVECs incubated with TCDD (40 nM), the COX‐2 inhibitor, NS‐398 (20 μM), the COX‐1 inhibitor, SC‐560 (20 μM), TCDD + NS‐398 or TCDD + SC‐560. *P < 0.05 *versus* vehicle, ^#^ *P* < 0.05 *versus* TCDD; *n* = 3. (**C**) Annexin V‐FITC/PI staining of HUVECs treated with TCDD (40 nM), NS‐398 (20 μM), SC‐560 (20 μM), TCDD + NS‐398 or TCDD + SC‐560 was assessed by flow cytometry. The percentages of early or late apoptotic cells are presented in the lower right and upper right quadrants, respectively. (**D**) Columns represent the proportions of apoptotic cells. *P < 0.05 *versus* vehicle, ^#^ *P* < 0.05 *versus* TCDD; *n* = 8–9. (**E**) Caspase 3 activity in HUVECs was measured using a Caspase 3 Activity Assay Kit. The treatments of HUVECs were the same as those described in Figure 1. *P < 0.05 *versus* vehicle, ^#^ *P* < 0.05 *versus* TCDD; *n* = 3.

COX‐1 and COX‐2 are the key enzymes in PG biosynthesis. COX‐2 protein expression was increased in HUVECs exposed to TCDD, whereas COX‐1 protein levels were not markedly altered (Fig. 2A). TCDD also increased mRNA levels of CYP1A1 and COX‐2, which peaked 4–8 hrs after TCDD challenge (*P* < 0.05; Fig. S1A and B).

TCDD can stimulate COX‐2 expression through both a non‐genomic pathway 31 or activation of the AhR/ARNT heterodimeric transcription factor 32. We further investigated whether TCDD‐caused COX‐2 expression in ECs occurs through direct activation of the AhR/ARNT transcription complex. We observed that increases in COX‐2 mRNA and protein expression after TCDD treatment were abolished in HUVECs by knockdown of the ARNT gene (Fig. S1D and E), indicating that TCDD caused COX‐2 expression in ECs through binding to the AhR and activation of the AhR/ARNT transcription complex. In addition, TCDD significantly reduced HUVEC viability (*P* < 0.05, Fig. 2B), this decline was abrogated by a selective COX‐2 inhibitor, NS‐398 (*P* < 0.05), but not by the COX‐1 inhibitor SC‐560 (Fig. 2B). Moreover, NS‐398 also reduced the increased apoptosis (*P* < 0.05; Fig. 2C and D) and caspase 3 activity (*P* < 0.05; Fig. 2E) caused by TCDD treatment. Thus, these results suggest that COX‐2 is involved in TCDD‐caused HUVEC apoptosis.

### COX‐2‐derived PGE_2_ facilitates TCDD‐caused apoptosis in HUVECs *via* the EP3 receptor

**Figure 3:**
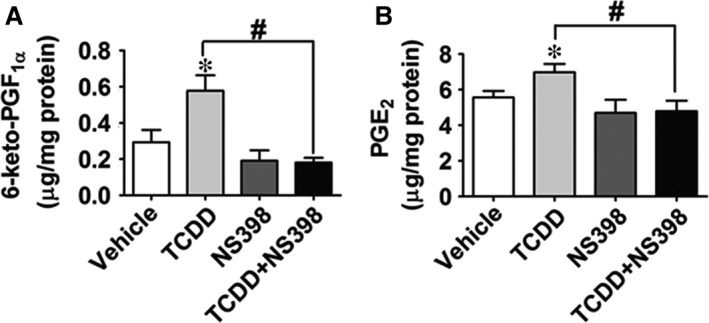
PGE_2_ and PGI_2_ production are increased in human umbilical vein endothelial cells (HUVECs) treated with 2, 3, 7, 8‐tetrachlorodibenzo‐*p*‐dioxin (TCDD). (**A**) and (**B**) PGE_2_ and 6‐keto‐PGF_1α_ (stable hydrolyzed product of PGI_2_) levels in HUVEC culture medium were determined by LC/MS/MS. *P < 0.05 *versus* vehicle, ^#^ *P* < 0.05 *versus* TCDD; *n* = 8–9.

**Figure 4:**
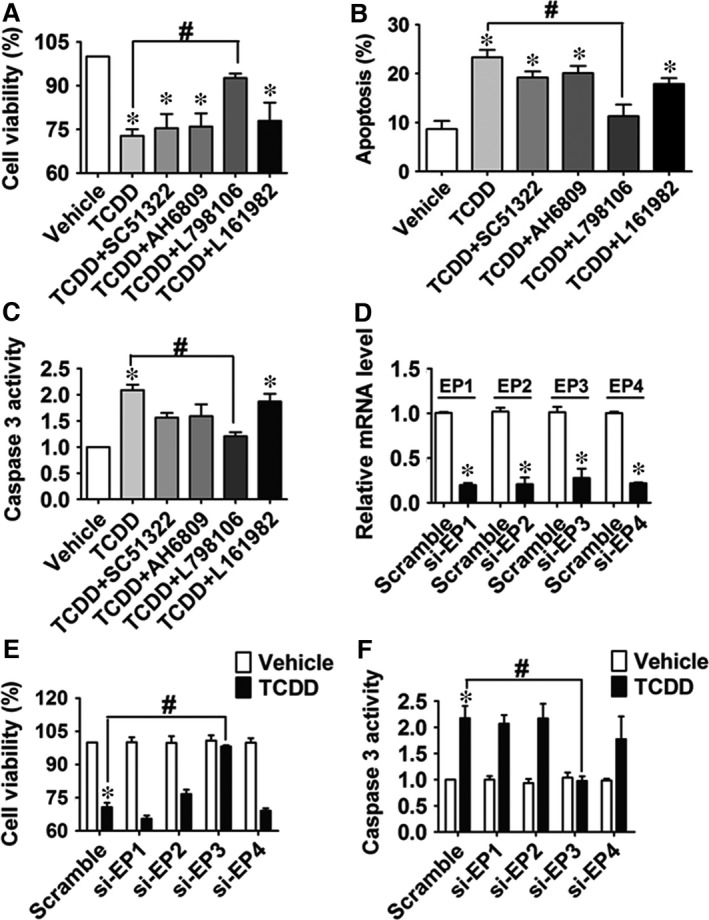
Blocking or knocking down of the EP3 receptor attenuates 2, 3, 7, 8‐tetrachlorodibenzo‐*p*‐dioxin (TCDD)‐caused apoptosis in human umbilical vein endothelial cells (HUVECs). (**A–C**) Cell viability, apoptosis and caspase 3 activity of HUVECs were analysed as described above. HUVECs were treated with EP1‐4 antagonists (10 μM SC51322, AH6809, L798106 or L161982,) for 24 hrs and then incubated with 40 nM TCDD for an additional 24 hrs. *P < 0.05 *versus* vehicle, ^#^ *P* < 0.05 *versus* TCDD; *n* = 3. (**D**) The knockdown efficiency of the EP1‐4 receptors was measured by qRT‐PCR. HUVECs were transfected with EP1‐4 siRNA or scrambled siRNA, and EP1‐4 mRNA levels were determined. *P < 0.05 *versus* scrambled siRNA; *n* = 4. (**E–F**) HUVECs were transfected with EP1‐4 siRNA or scrambled siRNA and then incubated with TCDD (40 nM) or DMSO for 24 hrs, cell viability (**E**) and caspase 3 activity (**F**) of HUVECs were then assessed. *P < 0.05 *versus* scrambled siRNA + vehicle, ^#^ *P* < 0.05 *versus* scrambled siRNA + TCDD; *n* = 3‐4.

We next assessed PG production in TCDD‐treated HUVECs and found that TCDD‐caused increases of COX‐2 expression in HUVECs were associated with a higher production of PGE_2_ and PGI_2_ (measured as its stable hydrolyzed product, 6‐keto‐PGF_1α_) in culture, which could be blocked by NS‐398 (*P* < 0.05; Fig. 3A and B). However, production of other PGs (PGD_2_, PGF_2α_ and TxB_2_) was not markedly altered by treatment with TCDD or the COX‐2 inhibitor NS‐398 (Fig. S2A–C). Expression of PGI_2_ receptor‐the IP was unchanged in HUVECs after TCDD exposure (Fig. S3A), and IP antagonist CAY10441 had no significant effects on the increased caspase 3 activity and reduced viability of TCDD‐treated HUVECs (Fig. S3B and C). To further identify which PGE_2_ receptor mediates HUVEC apoptosis caused by TCDD, both pharmacological inhibitors and specific siRNAs targeting EP1‐4 receptors were used to pre‐treat HUVECs followed by TCDD stimulation. As shown in Figure 4, TCDD‐caused apoptosis in HUVECs, evidenced by cell viability and caspase 3 activity measurements, was significantly attenuated by the EP3 inhibitor, L798106 (*P* < 0.05; Fig. 4A–C) and EP3 siRNA (*P* < 0.05, Fig. 4D–F), suggesting that the EP3 receptor plays a pivotal role in TCDD‐mediated apoptosis in ECs. In addition, we determined the transcript levels of EP1‐4 in HUVECs exposed to TCDD and found that only EP3 mRNA expression was elevated notably after 40 nM TCDD challenge (*P* < 0.05; Fig. S4A–D).

### The EP3‐dependent p38MAPK/Bcl‐2 pathway is activated in TCDD‐treated HUVECs

**Figure 5:**
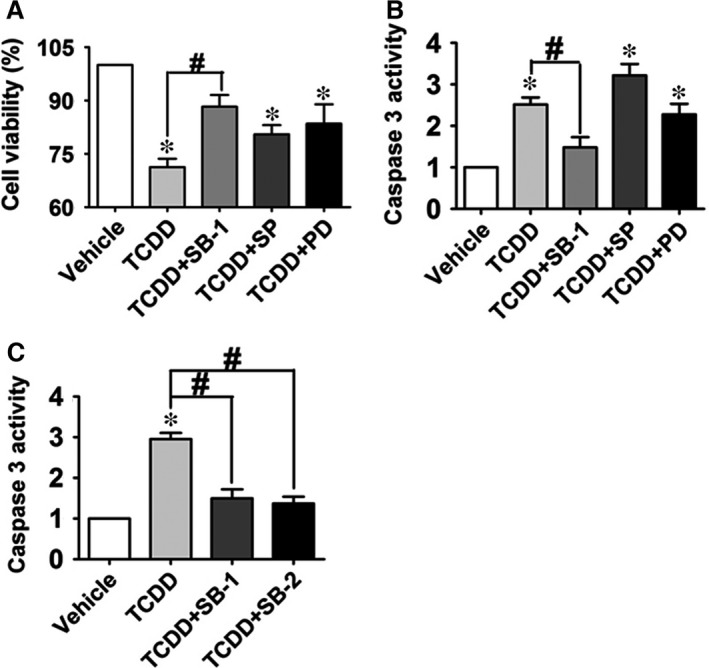
Inhibition of p38 MAPK signalling pathway diminishes human umbilical vein endothelial cells (HUVEC) apoptosis caused by 2, 3, 7, 8‐tetrachlorodibenzo‐*p*‐dioxin (TCDD). (**A**) and (**B**) HUVECs were treated with TCDD or combined with the ERK inhibitor, PD98059 (PD, 30 μM), the JNK inhibitor, SP600125 (SP, 20 μM) or the p38MAPK inhibitor, SB203580 (SB‐1, 20 μM) for 12 hrs, respectively, then stimulated with 40 nM TCDD for an additional 24 hrs. Cell viability and caspase 3 activity of HUVECs were measured. *P < 0.05 *versus* vehicle, ^#^ *P* < 0.05 *versus* TCDD; *n* = 3. (**C**) Effects of two different p38MAPK inhibitors (SB‐1 and SB202190, SB‐2) on caspase 3 activity in HUVECs treated with TCDD. *P < 0.05 *versus* vehicle, ^#^ *P* < 0.05 *versus* TCDD; *n* = 3.

**Figure 6:**
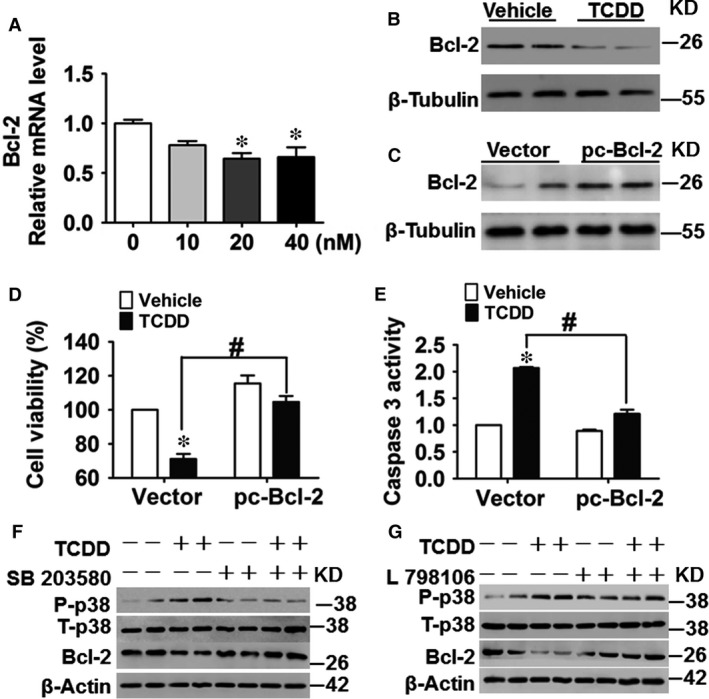
The EP3‐dependent p38MAPK/Bcl‐2 pathway is activated in 2, 3, 7, 8‐tetrachlorodibenzo‐*p*‐dioxin (TCDD)‐treated human umbilical vein endothelial cells (HUVECs). (**A**) The mRNA levels of Bcl‐2 in TCDD‐treated HUVECs were determined by qRT‐PCR. TCDD dose is shown in the *X*‐axis. *P < 0.05 *versus* the 0 nM group; *n* = 3. (**B**) Protein levels of Bcl‐2 in HUVECs treated with or without 40 nM TCDD for 24 hrs were analysed by Western blot. (**C–E**) HUVECs were transfected with pcDNA3.1‐Bcl‐2 (pc‐Bcl2) plasmid or vector and then incubated with TCDD (40 nM) or DMSO for 24 hrs. Cells were harvested for Bcl2 protein expression analysis (**C**), cell viability (**D**) and caspase 3 activity assays (**E**). *P < 0.05 *versus* vector + vehicle, ^#^ *P* < 0.05 *versus* vector + TCDD; *n* = 3. (**F–G**) HUVECs were pre‐treated with the ERK inhibitor, SB203580 (**F**) or L798106 (**G**) for 12 hrs, respectively, and then stimulated with 40 nM TCDD for an additional 24 hrs. Protein levels of phosphorylated p38 MAPK, total p38 MAPK and Bcl‐2 were examined by Western blot.

MAPK is involved in regulating the proliferation, survival and cell death responses of many cell types 33, 34. To explore whether intracellular JNK, p38 MAPK and ERK signalling pathways were involved in enhanced cell apoptosis in response to TCDD exposure, a series of experiments were performed using the MAPK subtypes inhibitors. Among them, the p38 MAPK inhibitor, SB203580, abrogated TCDD caused HUVEC apoptosis (*P* < 0.05; Fig. 5A and B). HUVEC viability was reduced ~30% by TCDD exposure, which was restored by treatment with SB203580 (*P* < 0.05; Fig. 5A). Meanwhile, TCDD stimulation elevated caspase 3 activity in HUVECs to approximately 2.5‐fold of the control (*P* < 0.05), which was diminished by SB203580 treatment (*P* < 0.05; Fig. 5B). The similar results were observed for another p38 MAPK inhibitor SB202190 (Fig. 5C), indicating that TCDD treatment results in HUVEC apoptosis *via* p38 MAPK signalling. Apoptosis is regulated precisely by the interaction between pro and anti‐apoptotic Bcl‐2 family proteins. Primary anti‐apoptotic proteins include Bcl‐2, Bcl‐XL and Mcl‐1, while pro‐apoptotic proteins are composed of Bax, Bak, Bim, Bid, Bad and Bik 35. We therefore measured the mRNA levels of Bad, Bak1, Bax, Bcl‐2 and Bim and found that only Bcl‐2 transcript levels decreased in TCDD‐treated HUVECs (*P* < 0.05; Figure 6A and Fig. S5A–D). Similarly, Bcl‐2 protein expression also was greatly inhibited by TCDD treatment as compared to the vehicle (Fig. 6B). In addition, reduction in cell viability and elevation of caspase 3 activity by TCDD treatment were reversed completely in HUVECs by exogenous Bcl‐2 overexpression (*P* < 0.05; Fig. 6C–E). Then, we examined whether Bcl‐2 is the downstream target gene of the EP3/p38MAPK axis, which regulates TCDD‐caused apoptosis. We found that TCDD promoted p38 MAPK phosphorylation and decreased Bcl‐2 protein expression, while the p38 inhibitor SB203580 recovered Bcl‐2 expression levels (Fig. 6F). Furthermore, EP3 inhibition by L798106 suppressed the augmented p38 MAPK phosphorylation and subsequently restored Bcl‐2 protein expression in TCDD‐treated HUVECs (Fig. 6G), further supporting activation of EP3/p38MAPK/Bcl‐2‐mediated TCDD‐triggered apoptosis in HUVECs.

### Activated RhoA is required for EP3‐mediated apoptosis of HUVECs caused by TCDD

**Figure 7:**
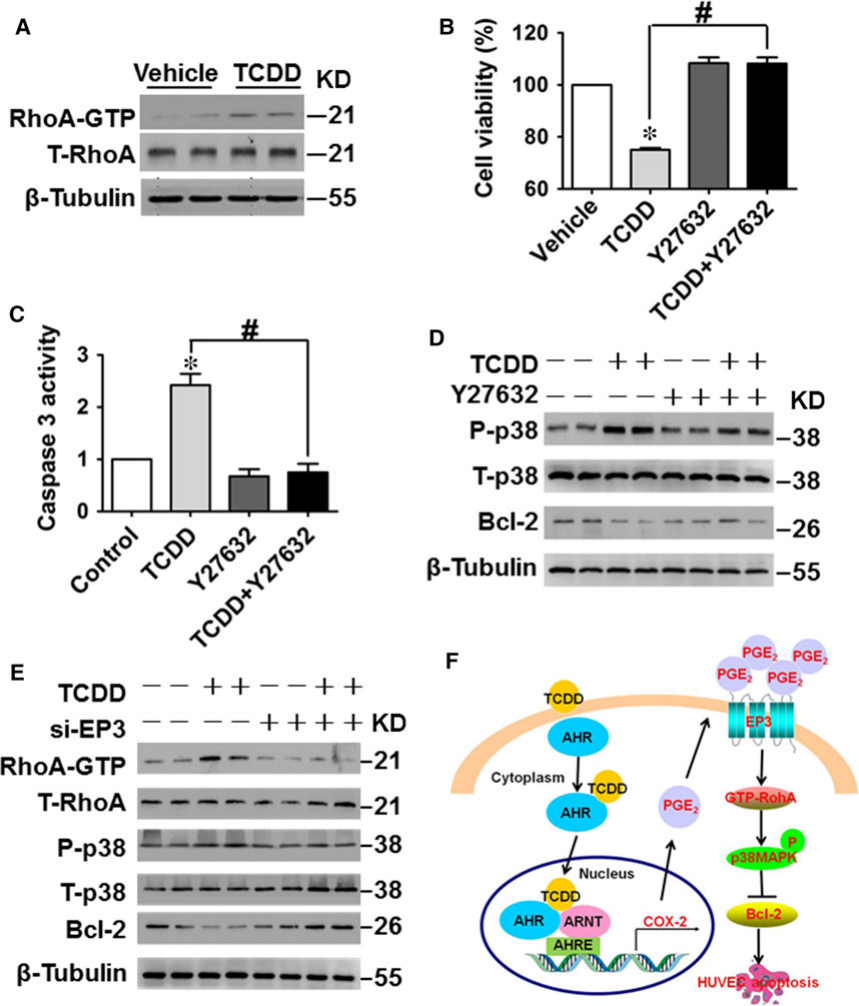
Activated RhoA is required for EP3‐mediated apoptosis in human umbilical vein endothelial cells (HUVECs) caused by 2, 3, 7, 8‐tetrachlorodibenzo‐*p*‐dioxin (TCDD). (**A**) Effects of TCDD on RhoA protein levels in HUVECs. HUVECs were treated with 40 nM TCDD for 3 min. (**B–C**) HUVECs were treated with the ROCK inhibitor, Y27632 (20 μM) for 12 hrs and then stimulated with 40 nM TCDD for an additional 24 hrs. Cell viability (**B**) and caspase 3 activity (**C**) of HUVECs were measured. *P < 0.05 *versus* vehicle, ^#^ *P* < 0.05 *versus* TCDD; *n* = 3. (**D**) Phosphorylated p38 MAPK, total p38 MAPK and Bcl‐2 expression levels were analysed by Western blot. (**E**) Protein expression levels of RhoA GTP, total RhoA, phosphorylated p38 MAPK, total p38 MAPK and Bcl‐2 in HUVECs transfected with EP3 siRNA or negative control siRNA with or without TCDD stimulation. (**F**) Schematic diagram of TCDD‐caused HUVEC apoptosis through affecting the COX‐2/PGE_2_/EP3/RhoA/p38MAPK/Bcl‐2 pathway.

As the EP3 receptor is linked to the small GTP‐binding protein Rho 24, 28, and Rho activates the p38 MAPK pathway 36, 37, 38, we investigated whether Rho mediates p38 activation in TCDD‐elicited apoptosis in HUVECs. Non‐radioactive Rho pull‐down assay showed that RhoA activity was minimal in unstimulated HUVECs, but was greatly increased following TCDD treatment (Fig. 7A). Interestingly, Rho‐associated protein kinase (ROCK) blocker Y26732 attenuated the reduction in cell viability and activation of caspase 3 in HUVECs following TCDD challenge (*P* < 0.05; Fig. 7B and C). In addition, Y27632 restored the altered p38 MAPK phosphorylation levels and Bcl‐2 protein levels in TCDD‐treated HUVECs (Fig. 7D), implying that RhoA/p38MAPK/Bcl‐2 signalling participates in apoptosis in HUVECs caused by TCDD. Furthermore, EP3 receptor knockdown suppressed activation of RhoA activity and p38MAPK phosphorylation and restored the reduced Bcl‐2 protein levels in TCDD‐treated HUVECs (Fig. 7E). Taken together, we conclude that TCDD promotes HUVEC apoptosis through the COX‐2/PGE_2_/EP3/RhoA/p38MAPK/Bcl‐2 pathway (Fig. 7F).

### TCDD exposure suppresses re‐endothelialization of wire‐injured femoral arteries by activating the EP3 receptor in mice

**Figure 8:**
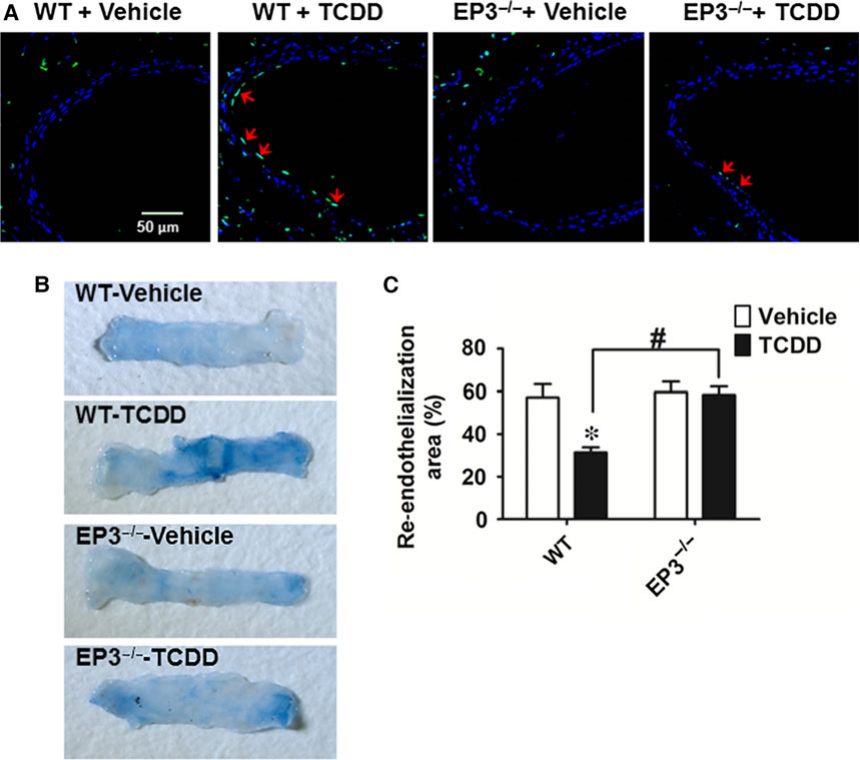
EP3 deficiency protects against 2, 3, 7, 8‐tetrachlorodibenzo‐*p*‐dioxin (TCDD)‐caused endothelial cell apoptosis in mice. Femoral artery injuries were induced by wire insertion in EP3^−/−^ and WT mice, TCDD (10 μg/kg body weight) was injected to mice intraperitoneally every other day, and the injured femoral arteries were collected on day 10 for TUNEL assay and Evans blue dye staining. (**A**) Representative confocal microscope images of TUNEL‐stained femoral arteries. Blue indicates DAPI staining, and green indicates TUNEL staining. Arrows indicate TUNEL‐positive cells. Scale bar, 50 μm. (**B**) Representative photomicrographs of femoral arteries stained by Evans blue dye on day 10 after injury. The area stained blue corresponds to the area not yet re‐endothelialized. (**C**) Quantification of the re‐endothelialized area assessed by percentage of the area unstained with Evans blue dye in the entire injured area. *P < 0.05 *versus* WT + vehicle; *n* = 9–10.

To further test whether the EP3 receptor participates in apoptosis and re‐endothelialization *in vivo*, WT and the EP3 receptor knockout mice were injured in the femoral arteries and injected intraperitoneally with TCDD. Then, the apoptosis and re‐endothelialization in injured vessels were examined using a TUNEL kit and Evans blue staining, respectively. Ten days after the injury, TCDD injection resulted in more apoptosis in the vascular endothelium of WT mice than that of EP3^−/−^ mice (Fig. 8A). In agree with apoptosis, the degree of re‐endothelialization in WT mice, but not in EP3^−/−^ mice, was significantly lower after TCDD injection than after the vehicle injection (*P* < 0.05; Fig. 8B and C).

## Discussion

TCDD elicits a broad spectrum of toxic and biochemical responses including a wasting syndrome, tumour promotion, teratogenesis, hepatotoxicity, modulation of endocrine systems, immunotoxicity and cardiovascular diseases 39, 40. Many of these effects are mediated *via* the AhR activation 41. Epidemiological and animal studies have shown that TCDD exposure contributes to pathogenesis of cardiovascular diseases 11, 13, 42. In this study, we found that TCDD promoted endothelial apoptosis through the COX‐2/PGE_2_/EP3/p38MAPK/Bcl‐2 pathway and impaired vascular re‐endothelialization after injury in mice through the EP3 receptor.

COX catalyses the first step in the formation of PGs from AA. Two isoforms of COX are often referred to as COX‐1 and COX‐2. Notably, COX‐2 plays an important role in the regulation of cell survival and proliferation. In human osteosarcoma cells, overexpression of the COX‐2 gene has been shown to suppress proliferation and promote apoptosis 43. High glucose levels induce COX‐2 expression and facilitate apoptosis in HUVECs 20. Similarly, we observed that TCDD treatment increased HUVEC apoptosis in a dose‐dependent manner by up‐regulating COX‐2 expression. However, COX‐2 promotes cell proliferation and survival in some cancer cell lines 44, 45, 46. These discrepancies may be ascribed to different functions of multiple PGs and receptors downstream of COXs 47, 48. There are four subtypes of the PGE_2_ receptor (EP1‐4). We found that TCDD caused HUVEC apoptosis by activating the COX‐2/PGE_2_/EP3 axis. Similarly, the EP3 receptor also mediates apoptosis in neurons in the murine ischemic cortex 49.

The MAPK family contains JNK, p38 MAPK and ERK. Extensive studies demonstrated a critical role for MAPK signalling pathways in the regulation of various cellular processes, including migration, proliferation, differentiation, development, apoptosis and cell cycle arrest 50. We assessed whether intracellular JNK, p38 MAPK and ERK signal pathways were involved in enhanced cell apoptosis in response to TCDD exposure and revealed that only the p38 MAPK inhibitor, SB203580, attenuated TCDD‐caused apoptosis in HUVECs. The increased p38 MAPK phosphorylation by TCDD exposure was blocked by SB203580. Several studies have shown that TCDD‐caused apoptosis depends on activation of different MAPK pathways in various cell types. In the RAW 264.7 cells, both p38 MAPK and ERK pathways mediate TCDD‐caused apoptosis 51. However, JNK and ERK signalling are activated in TCDD‐caused Jurkat T lymphocyte cell apoptosis 52. Li *et al*., 34 demonstrated that TCDD could lead to apoptosis in rat primary cortical neurons through mediating p38 MAPK and JNK pathways.

The Bcl‐2 protein family regulates apoptosis and contains both anti‐apoptotic (Bcl‐2, Bcl XL, and Mcl 1) and pro‐apoptotic proteins (Bax, Bak, Bim, Bid, Bad and Bik) 35. We found that Bcl‐2 mRNA and protein expression were markedly reduced in TCDD‐treated HUVECs. Forced expression of the Bcl‐2 gene attenuated TCDD‐caused apoptosis of HUVECs, along with suppression of caspase 3 activity. In agreement with our observations, TCDD also promotes apoptosis of human trophoblast‐like JAR cells 53 and bovine MDBK cells 54 by down‐regulating expression of the Bcl‐2 gene. Given that EP3 inhibition or knockdown restored TCDD‐caused‐p38 MAPK activation and suppressed Bcl‐2 expression, EP3‐mediated activation of the p38MAPK/Bcl‐2 pathway contributes to apoptosis increased by TCDD in HUVECs. Rho activates p38 MAPK 36, 37, 38 and the EP3 receptor couples small G protein and activates RhoA 24, 28. Indeed, we demonstrated that TCDD exposure increased p38 MAPK phosphorylation by activating EP3/Rho signalling in HUVECs.

In summary, TCDD exposure increases endothelial apoptosis through the COX‐2/PGE_2_/EP3/p38MAPK/Bcl‐2 pathway and delays arterial re‐endothelialization after injury through activation of the EP3 receptor. These findings suggest that blocking the EP3 receptor may confer cardiovascular protection against TCDD toxicity.

## Author contributions

Yu Yu and Ying Yu conceived and designed the research; Yu Yu, Qian Liu, Shumin Guo, Qianqian Zhang, Juan Tang, Guizhu Liu, Deping Kong, Juanjuan Li and Shuai Yan performed the experiments; Yu Yu and Qian Liu wrote the manuscript; Yu Yu, Ruiguo Wang, and Peilong Wang analysed the data; Yu Yu, Xiaoou Su and Ying Yu revised the manuscript.

## Conflicts of interest

The authors report that there are no conflicts of interest.

## Supporting information


**Fig. S1** Effect of ARNT knockdown on COX‐2 expression in TCDD‐treated HUVECs.

**Fig. S2** PGD_2_, PGF_2α_ and TxB_2_ production in HUVECs treated by TCDD.

**Fig. S3** PGI_2_ receptor (IP) inhibition has no effect on TCDD‐caused endothelial cell apoptosis.

**Fig. S4** The mRNA levels of PGE_2_ receptors in HUVECs treated by TCDD.

**Fig. S5** mRNA levels of mitochondrial apoptotic genes in HUVECs treated by TCDD.

**Table S1** Primers used in real‐time quantitative PCR.
