# Supplementary figures and images for: 2, 3, 7, 8‐Tetrachlorodibenzo‐p‐dioxin promotes endothelial cell apoptosis through activation of EP3/p38MAPK/Bcl‐2 pathway
Source: J Cell Mol Med. 2017 Jul 12;21(12):3540–51. doi: 10.1111/jcmm.13265 (PMC5706494; doi:10.1111/jcmm.13265)

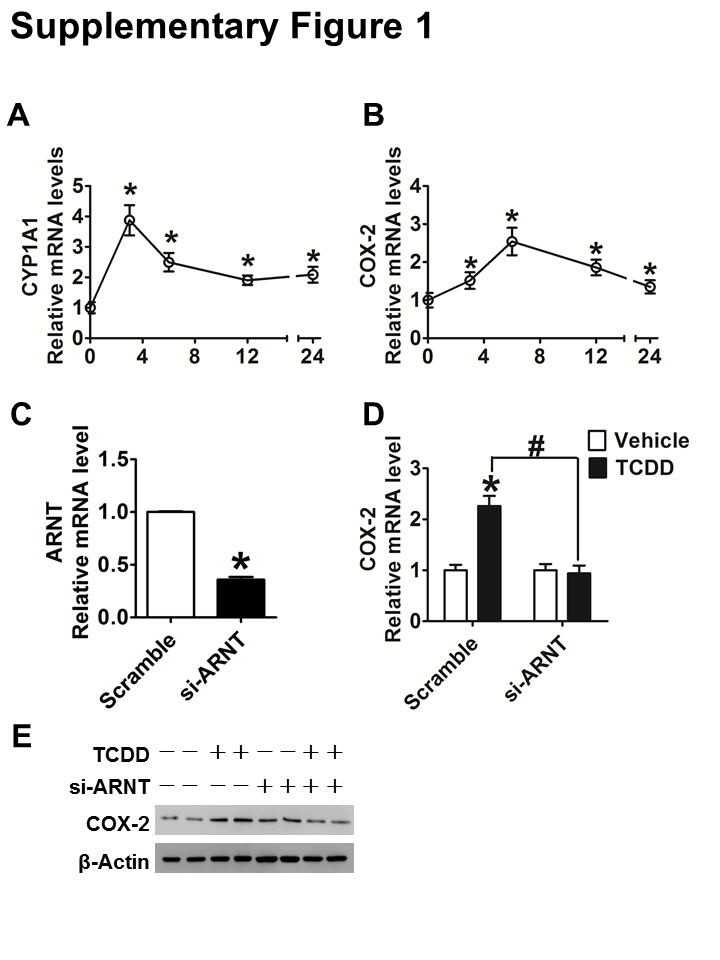

Supplement: Supplementary file 1 — Fig. S1 Effect of ARNT knockdown on COX‐2 expression in TCDD‐treated HUVECs. [file JCMM-21-3540-s001.TIFF]

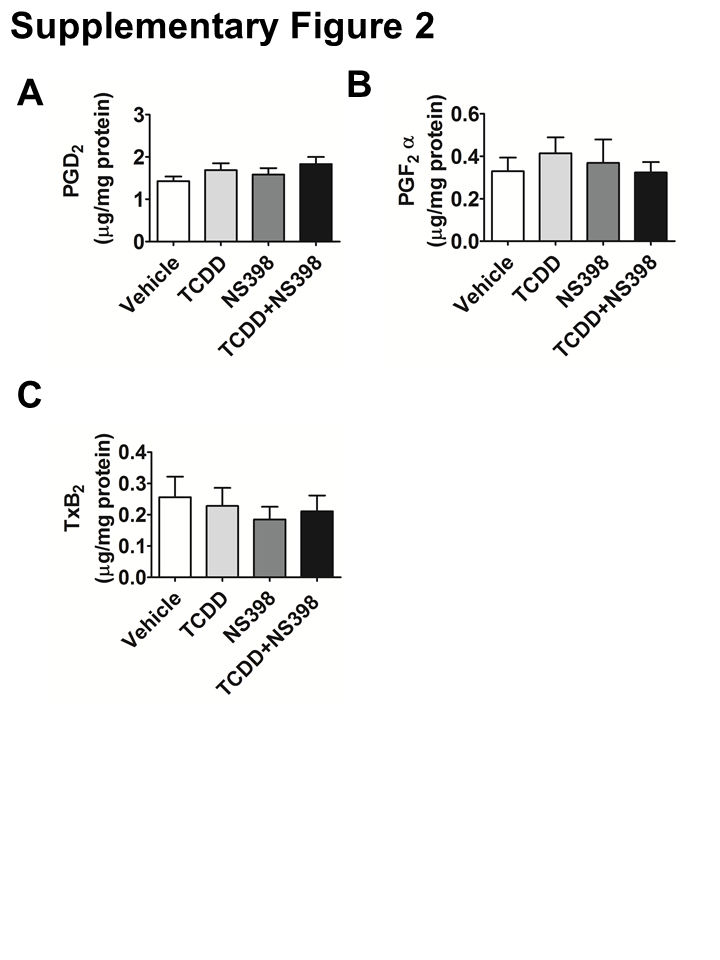

Supplement: Supplementary file 2 — Fig. S2 PGD2, PGF2α and TxB2 production in HUVECs treated by TCDD. [file JCMM-21-3540-s002.TIFF]

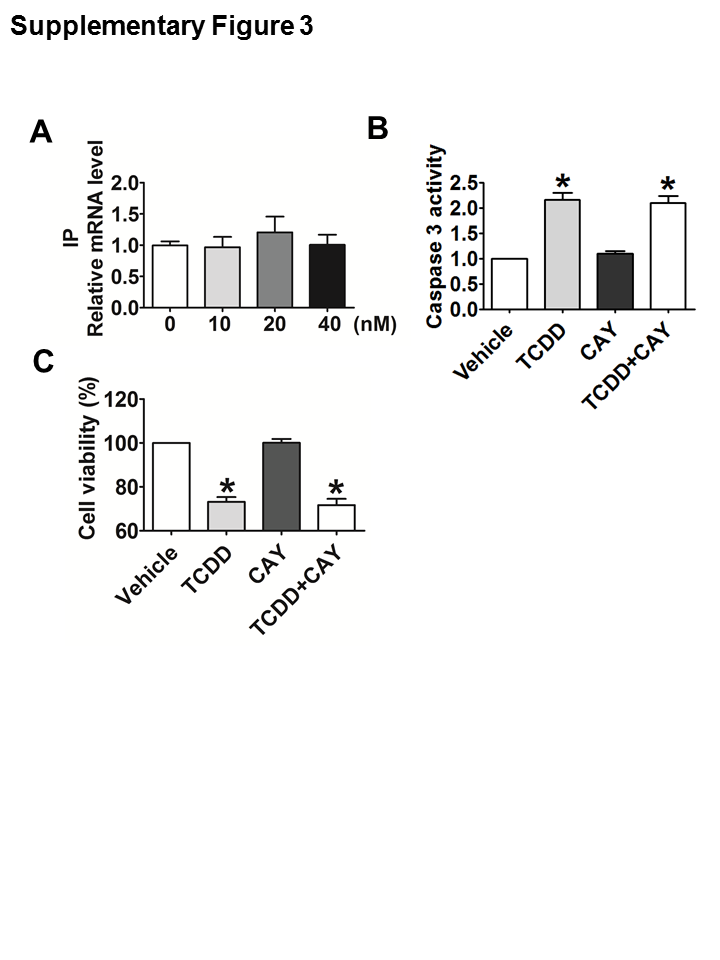

Supplement: Supplementary file 3 — Fig. S3 PGI2 receptor (IP) inhibition has no effect on TCDD‐caused endothelial cell apoptosis. [file JCMM-21-3540-s003.TIFF]

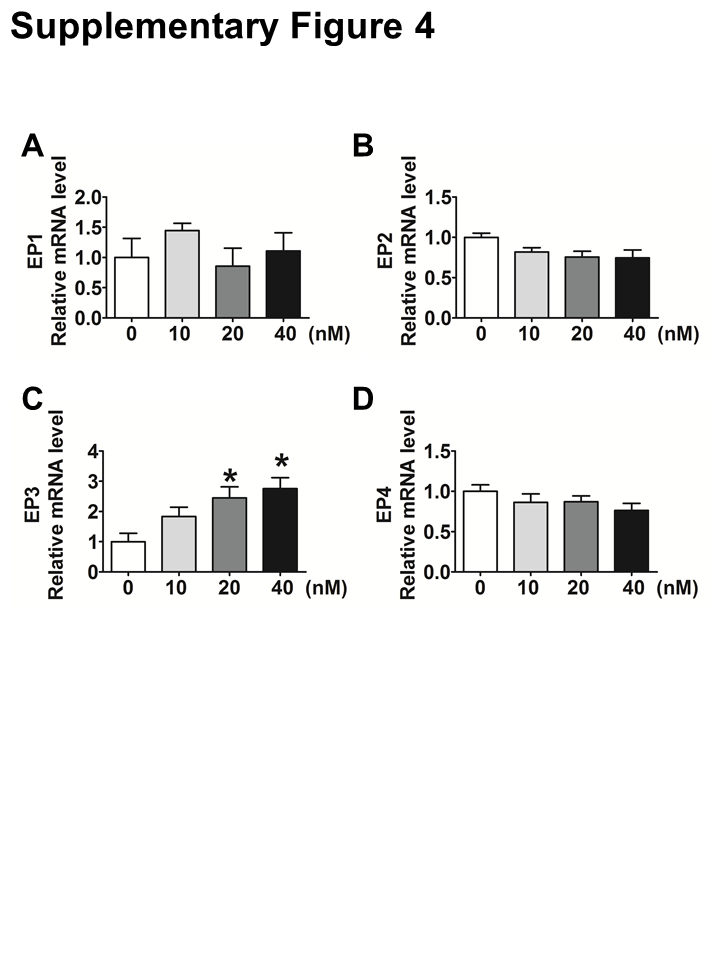

Supplement: Supplementary file 4 — Fig. S4 The mRNA levels of PGE2 receptors in HUVECs treated by TCDD. [file JCMM-21-3540-s004.TIFF]

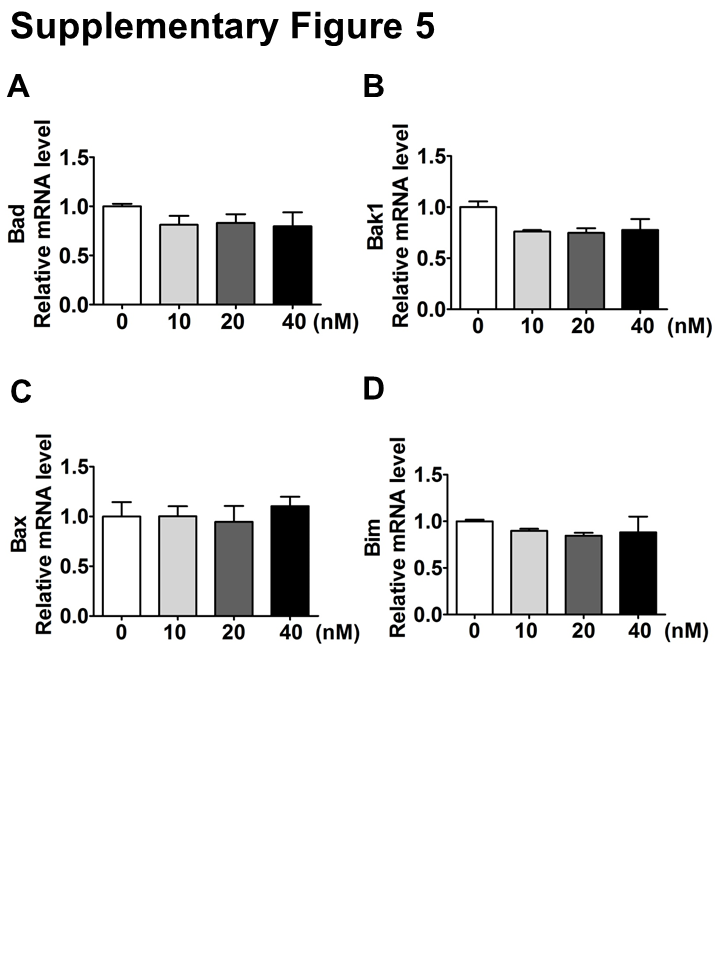

Supplement: Supplementary file 5 — Fig. S5 mRNA levels of mitochondrial apoptotic genes in HUVECs treated by TCDD. [file JCMM-21-3540-s005.TIFF]
