# Supplementary material for: 2, 3, 7, 8‐Tetrachlorodibenzo‐p‐dioxin promotes endothelial cell apoptosis through activation of EP3/p38MAPK/Bcl‐2 pathway
Source: J Cell Mol Med. 2017 Jul 12;21(12):3540–51. doi: 10.1111/jcmm.13265 (PMC5706494; doi:10.1111/jcmm.13265)
Supplement: Supplementary file 6 — Table S1 Primers used in real‐time quantitative PCR. [file JCMM-21-3540-s006.docx]

| **Supplementary Table 1 Primers used in real-time quantitative PCR** | | |
| --- | --- | --- |
| **Gene name** | **Forward primer sequence (5'-3')** | **Reverse primer sequence (5'-3')** |
| GAPDH | CATGAGAAGTATGACAACAGCCT | AGTCCTTCCACGATACCAAAGT |
| EP1 | AGCTTGTCGGTATCATGGTG | AAGAGGCGAAGCAGTTGGC |
| EP2 | CGATGCTCATGCTCTTCGC | GGGAGACTGCATAGATGACAGG |
| EP3 | CGCCTCAACCACTCCTACAC | GACACCGATCCGCAATCCTC |
| EP4 | CCGGCGGTGATGTTCATCTT | CCCACATACCAGCGTGTAGAA |
| IP | TTCCGCTTCTACGCCTTCAAC | ACCCAGAGCTTGAGTCGCT |
| Bad | CACCAGCAGGAGCAGCCAAC | CGACTCCGGATCTCCACAGC |
| Bak1 | GTTTTCCGCAGCTACGTTTTT | GCAGAGGTAAGGTGACCATCTC |
| Bax | CCCGAGAGGTCTTTTTCCGAG | CCAGCCCATGATGGTTCTGAT |
| Bcl-2 | TTGACAGAGGATCATGCTGTACTT | TCAGTCTACTTCCTCTGTGATGTTGT |
| Bim | TGTCTGACTCTGACTCTCTGACTGA | GAAGGTTGCTTTGCCATTTGGTC |
| ARNTsiRNA | GGCAGAGAAUUUCAGGAAUTT | AUUCCUGAAAUUCUCUGCCTT |
| EP1siRNA | GCCAGCUUGUCGGUAUCAUTT | AUGAUACCGACAAGCUGGCTT |
| EP2siRNA | CCUGUCAUCUAUGCAGUCUTT | AGACUGCAUAGAUGACAGGTT |
| EP3siRNA | GCUUCACUGAACCAGAUCUTT | AGAUCUGGUUCAGUGAAGCTT |
| EP4siRNA | CCAGUUAUAUCAGCCAAGUTT | ACUUGGCUGAUAUAACUGGTT |
