# Supplementary material for: 2, 3, 7, 8‐Tetrachlorodibenzo‐p‐dioxin promotes endothelial cell apoptosis through activation of EP3/p38MAPK/Bcl‐2 pathway
Source: J Cell Mol Med. 2017 Jul 12;21(12):3540–51. doi: 10.1111/jcmm.13265 (PMC5706494; doi:10.1111/jcmm.13265)
Supplement: Supplementary file 7 [file JCMM-21-3540-s007.docx]

**Supplementary Figure 1 Effect of ARNT knockdown on COX-2 expression in TCDD-treated HUVECs.**

mRNA levels of CYP1A1 **(A)** and COX-2 **(B)** in HUVECs exposed to TCDD for 0–24 hrs. * *P* < 0.05 *versus* the control group; n = 3. **(C)** The knockdown efficiency of ARNT was measured by qRT-PCR. * *P* < 0.05 *versus* scramble; n = 4. HUVECs were transfected with ARNT siRNA or negative control siRNA, then incubated with TCDD (40 nM) or DMSO for an additional 24 hrs for mRNA extraction or 48 hrs for protein extraction, respectively. COX-2 mRNA (**D**) and protein levels (**E**) in TCDD-treated HUVECs were analyzed. * *P* < 0.05 *versus* scramble+vehicle, ^#^ *P* < 0.05 *versus* scramble+TCDD; n = 6.

**Supplementary Figure 2 PGD_2_, PGF_2α_ and TxB_2_ production** **in HUVECs treated by TCDD.**

**(A-C)** Levels of PGD_2_, PGF_2α_ and TxB_2_ in HUVEC culture medium were determined by LC/MS/MS. * *P* < 0.05 *versus* vehicle, ^#^ *P* < 0.05 *versus* TCDD; n = 9.

**Supplementary Figure 3 PGI_2_ receptor (IP) inhibition has no effect on TCDD-caused endothelial cell apoptosis.**

**(A)** mRNA level of IP receptor in HUVECs. n = 3. (**B-C**) The effect of IP antagonist CAY10441 on cell viability **(B)** and caspase 3 activity **(C)** in TCDD-treated HUVECs. Cells were treated with CAY10441 (2 μM) in combination with TCDD (40 nM), the cell viability and caspase activity in HUVECs was assayed after 24 hrs. * P < 0.05 versus vehicle; n = 3.

**Supplementary Figure 4 The mRNA levels of PGE_2_ receptors in HUVECs treated by TCDD.**

**(A-D)** mRNA levels of EP1-4 receptors in HUVECs were assayed by qRT-PCR. The concentrations of TCDD used were 0, 10, 20, and 40 nM, respectively. * *P* < 0.05 *versus* the 0 nM group; n = 3.

**Supplementary Figure 5 mRNA levels of mitochondrial apoptotic** **genes** **in HUVECs treated by TCDD.**

**(A-D)** mRNA levels of Bad (**A**), Bak1 (**B**), Bax (**C**) and Bim (**D**) in TCDD –treated HUVECs were measured by qRT-PCR. n = 3.
